# The Plea of Impulse

**Published:** 1921-07-09

**Authors:** 


### THE PLEA OF IMPULSE.

The Hospital travels far. The writer has been reading on May 2 the April 2, 1921, number in the wilds of Kashmir. The article "Crime and Psycho-analysis" and the remembrance of former articles recently published have brought again the impulse to write, long stifled for want of leisure.

Surveyed from a distance and from a part of the world in which crime is called crime and punished, if detected, without great consideration of subtle excuses, British civilisation appears to be morbidly sentimental and too soft towards the criminal. Anything is an excuse to allow the murderer or the thief to escape chastisement. This is no very new attitude towards crime, all that is new is the attempt by psycho-analysis to add more to the excuses for it. The result of mercy and softness to the criminal does not seem to be satisfactory. To judge by much recently published, crime is rampant in Great Britain. A large section of the public does not regard theft as an act man or woman should be ashamed of committing. Notably this applies to the highly-paid railwaymen and dock-hands and transport workers in general. The depredations made on goods in transport amounts to millions a year, according to British publications quoted in Indian papers. It is practically impossible that the majority of the railwaymen, dockmen and so forth do not know which men amongst them are actively guilty of theft, even if an actual majority are not guilty; yet so few feel it a slur on their unions that they enfold many thieves that practically never, it appears, is information lodged, and no steps seem to be taken by the unions to ostracise the dishonest. Perhaps they are believers in irresistible impulse and so, in pity for the poor sufferers from it, they leave them to the consolation afforded by the illgotten gains of impulse. This is more satisfactory to the thieves than to the consignors or consignees of goods.

Murder is less on the increase. The irresistible impulse is apt to come up against the irresistible post and to swing on a beam attached to it; first offence cannot be pleaded for homicide, yet, though perhaps like the dog, in time the murderer in Great Britain will be allowed his first bite gratis. The sentimentalists would approve that without doubt.

The first-offence plea, or the consideration given to it, is a premium on dishonesty, almost an incentive to it. It relaxes the moral attitude towards theft. "After all," says the youth of criminal inclinations, "theft is not in itself a bad thing. You do not get punished for it unless you do too much of it." In most cases first offence is really first detection, and the narrow escape from gaol is a cause of caution, not of repentance. Honesty is a manufactured article, not an inherent attribute of man. It is an invention necessary to civilised societies. All men have by nature the impulse to steal, and it is made resistible by agreement of societies, which have decreed to free their members of the need constantly to watch and ward each his own possessions; to give right to possessions and punishment to those who breach the right. The punishments are the contempt and ostracism of the culprit's fellows, or such disciplinary action as gaol or the rod, or both. In such a state of society that contempt and social ostracism follow the detection of theft, irresistible impulse to take a neighbour's property is rare. But when there is neither contempt for nor ostracism of the thief theft flourishes. Hence it is rare in public schools in which contempt in full measure, expulsion, and social ruin follow the act; common in docks and on railways because the thief is not despised for dishonesty nor ostracised, though he may be twitted and despised should he go to gaol. The attitude of the village boy is not "Yah! Who's a thief?" but "Yah! Who went to prison?" No doubt it is not pleasant for little Tommy to do a little time or to feel a little birch, and possibly neither does him any good, but the unpleasantness to Tommy has a most excellent effect on little Dick and Harry, who are impressed with the fact that theft is something not to be done. and in time that impression may develop almost into an instinct. Perhaps you damaged the character of little Tommy, but you vastly improved the characters of little Dick and Harry, and probably little Tommy's character was not much good in any case. So on the whole gain; and less irresistible impulse.

And are the persons who cannot resist their impulses desirable members of societies? After all societies only exist because the members of them do resist impulses. It seems to follow that those who cannot resist impulse should be perpetually excluded from societies and not pitied and coddled and allowed to hand down to descendants their incapacity to resist. It would be interesting to see, if a plea of inability to resist meant not the brutality of the gallows but the quiet elimination of a lethal chamber, what the result would be on the expert evidence in a murder trial or in a shop-lifting case. Instead of expert after expert swearing to symptoms of incapacity to resist, we might see experts pointing at the strong will and calculating ability of the criminal. Crime lessened, so it is said, when the draconic code of a century or so ago was displaced by more merciful measures, and it is assumed this was cause and effect. But it is arguable that this severity had eliminated many of the weakly resistant, and that therefore less severity seemed sufficient, and that now the result of mildness is an increase of the weak but impulsive. That is a point of view. But more likely the lessening of crime was due to the certainty of punishment of some severity. This acted as a deterrent on all but the habitually criminal, educated the mass to a sense of honesty, and made it worth while to resist the natural impulse to steal. Now the deterrent is slighter, the risk is worth while, for, on detection, first a plea of "first offence" and after of "irresistible impulse" saves from all consequence of crime except the advantage of acquisition.

